# NRF2 Modulators of Plant Origin and Their Ability to Overcome Multidrug Resistance in Cancers

**DOI:** 10.3390/ijms252111500

**Published:** 2024-10-26

**Authors:** Piotr Wadowski, Michał Juszczak, Katarzyna Woźniak

**Affiliations:** 1 Department of Molecular Genetics, Faculty of Biology and Environmental Protection, University of Lodz, Pomorska 141/143, 90-236 Lodz, Poland; piotr.wadowski@edu.uni.lodz.pl (P.W.); michal.juszczak@biol.uni.lodz.pl (M.J.); 2 Doctoral School of Exact and Natural Sciences, University of Lodz, Banacha 12/16, 90-237 Lodz, Poland

**Keywords:** NRF2 activators, NRF2 inhibitors, multidrug resistance, cancer, polyphenols, alkaloids

## Abstract

Cancer is one of the most common causes of death in the world. Despite the fact that there are many types of therapies available, cancer treatment remains a major challenge. The main reason for the ineffectiveness of chemotherapy is the acquisition of multidrug resistance (MDR) by cancer cells. One of the factors responsible for the acquisition of MDR is the NRF2 transcription factor, which regulates the expression of proteins such as *HO-1*, *NQO1*, MRP1, MRP2, and GST. In normal cells, NRF2 is the first line of defense against oxidative stress, thereby preventing carcinogenesis. Still, its hyperactivation in cancer cells causes them to acquire MDR, which significantly reduces or eliminates the effectiveness of chemotherapy. Considering the important role NRF2 plays in the acquisition of MDR, its modulators and, above all, inhibitors are being sought after, including among compounds of plant origin. NRF2 inhibition may prove to be a key element of anticancer therapy. This review summarizes the current state of knowledge about plant NRF2 inhibitors and presents the effects of their use in overcoming MDR in cancer.

## 1. Introduction

**Figure 1:**
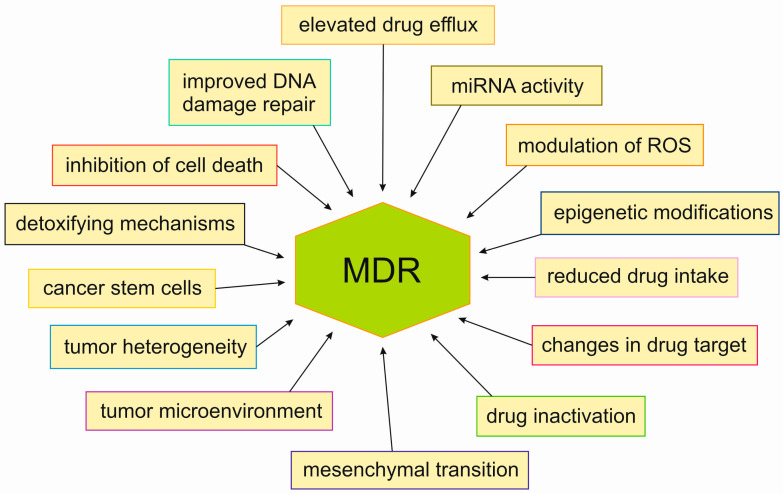
MDR acquisition strategies.

Multiple strategies for combating cancers have been developed throughout the years; despite this, there were 9.7 million deaths from cancer in the year 2022 worldwide. Moreover, there were close to 20 million new cases of cancer in that year [1]. One of the major factors leading to such a high mortality rate in cancers is the development of multidrug resistance (MDR), which makes the already difficult task of combating cancers even more complex. The most detrimental characteristic of MDR is that it leads to resistance to anticancer agents that are functionally and structurally different to those initially used in therapy. Cancer cells may acquire MDR through various strategies, such as elevated drug efflux, reduced drug intake, changes in drug targets and drug inactivation, epigenetic modifications, modulation of reactive oxygen species (ROS) levels and miRNA activity, improved DNA damage repair, inhibition of cell death, and detoxifying mechanisms, as well as the involvement of cancer stem cells, tumor heterogeneity, its microenvironment or mesenchymal transition (Figure 1) [2,3]. MDR cancer cells are characterized by their elevated antioxidant and scavenging enzyme levels, even when compared to other non-MDR cancers, which implies that the former may be far more susceptible to experiencing elevated ROS levels [4]. Thus, MDR is the main culprit behind inefficacy of various forms of cancer treatment and a critical subject for further studies regarding cancer treatment [5].

Nuclear factor erythroid 2-related factor 2 (NRF2), along with Kelch-like ECH-associated protein 1 (KEAP1) and antioxidant response elements (AREs), participates in the KEAP1/NRF2/ARE pathway, which regulates the cell’s cytoprotective actions [4]. It is also a transcription factor responsible for managing mitochondrial function, inflammation, and various cytoprotective actions like metabolism of xenobiotics and drugs [6,7,8]. Because of that, it is considered to be the main agent in maintaining redox balance at both the cellular and tissue levels, as it is responsible for regulating about 200 genes [7,9]. However, NRF2 activation is a double-edged sword, acting as a protector of both normal and cancer cells, depending on the circumstances of its activation. In normal cells, brief activation of NRF2, as a response to elevated ROS levels, acts as a cytoprotective, anticancer mechanism, as it reduces possible harm that might occur due to oxidative stress, which subsequently could lead to tumor initiation and progression [8,9]. On the other hand, in cancer cells, NRF2 tends to be overexpressed as a result of either constitutive activation or hyperactivation, allowing them to survive in ROS-rich environments (known as redox adaptation), which comes hand in hand with their increased proliferation and increased resistance to chemotherapy, which, a lot of the time, depends on ROS generation. So, in other words, NRF2 is one of the main factors responsible for MDR development in cancers [7,8].

## 2. Structure and Regulation of NRF2

**Figure 2:**
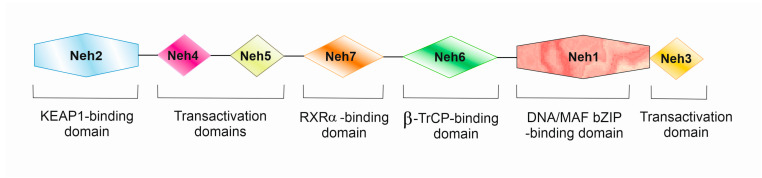
Domain structure of NRF2.

The NRF2 protein is a Cap“n”Collar (CNC), basic-region leucine zipper (bZIP) transcription factor [10]. Structural studies of the protein have shown the presence of seven highly conserved functional domains (Neh1–Neh7) (Figure 2). Neh1 has a leucine zipper domain that allows it to interact with DNA and small MAF (*v-Maf* avian musculoaponeurotic fibrosarcoma oncogene homolog) proteins. The Neh2 domain interacts with the negative KEAP1 regulator through specific DLG and ETG motifs and plays an important role in NRF2 degradation [11]. Neh3–Neh5 domains participate in NRF2 transactivation. The Neh3 domain enables transcription activation of genes containing the ARE motif. The Neh4 and Neh5 domains cooperatively bind to the cAMP response element-binding (CREB) protein (CBP), which allows them to perform a co-activating function for numerous transcription factors [12]. The Neh6 and Neh7 domains’ activity relates to post-translational regulation of NRF2. The Neh6 domain next to Neh2 is crucial in the degradation of NRF2 [13]. The Neh7 domain has the ability to interact with the retinoic X receptor α, resulting in NRF2 repression [14].

The main mechanisms of NRF2 regulation are based on post-transcriptional KEAP1-dependent regulation, post-transcriptional KEAP1-independent regulation, and transcriptional regulation. Under physiological conditions in the cell, NRF2 is present in cytosol, forming a complex with KAEP1, which is connected to an adapter—the Cullin 3-based ubiquitin E3 ligase complex (Cul3). Cul3 promotes the continuous ubiquitination of NRF2, which consequently leads to proteasomal degradation, keeping NRF2 protein at a low basal level [15]. Following cell exposure to oxidative stress, modification of cysteine residues of the KEAP1 protein occurs, inhibiting Cul3 enzymatic activity. Next, NRF2 is released from KEAP1 and begins to accumulate in the cell nucleus where it forms heterodimers with sMAF proteins. The heterodimer then binds to the AREs of the target genes, activating their transcription [16]. The mechanism of post-transcriptional regulation independent of KEAP1 is based on glycogen synthase kinase 3β (GSK-3β). GSK-3β may mediate ubiquitination and, consequently, proteasomal degradation of NRF2 by activating E3 ubiquitin ligase complex (β-TrCP-Skp1-Cul1-Rbx1). In addition, GSK may phosphorylate the serine moieties of the Neh6 domain, resulting in B-TrCP recognition associated with Cullin 1 (Cul1), subsequent ubiquitination, and proteasomal degradation [17].

## 3. Modulation of NRF2 as a Means to Combat MDR Cancers

NRF2 is often hyperactivated in MDR cancers, leading to increased expression of antioxidants such as heme oxygenase 1 (*HO-1*) and NAD(P)H quinone dehydrogenase 1 (*NQO1*), transporter proteins like multidrug resistance protein 1/2 (MRP1/2), and detoxifying enzymes like glutathione S-transferase (GST) [18]. This diversity of functionally distinct proteins, all of which are involved in MDR development, encourages us to look at NRF2 as a potential target for modulation, with the goal of sensitizing cancer cells to chemotherapy [2,7,19].

NRF2 modulators can be divided into two major groups depending on whether they increase or decrease their activity, each having its application in combating cancers. Activators of NRF2 could have potential use in cancer prevention, and reduce the danger of carcinogenesis occurring, while inhibitors could be used as a means to sensitize cancer cells to treatment and reverse MDR. One thing worth noting is that numerous NRF2 activators activate NRF2 in normal cells and inhibit it in cancer cells [20].

## 4. Natural Compounds as NRF2 Modulators

Due to unsatisfactory results regarding the use of synthetic compounds in combating MDR, researchers have shifted their interest towards natural compounds, some of which could potentially be used as NRF2-modulating agents [2]. Some polyphenols can prevent the development of MDR and oxidative stress, as well as inhibit angiogenesis, metastasis, proliferation, and survival of cancer cells, modulate inflammatory and immune responses, and even inactivate pro-oncogenes by induction of NRF2 [2,21]. Alkaloids, despite being well-known inhibitors of NRF2 activity, have been examined rather poorly [7]. Their inhibitory effect on NRF2 may have two different effects on cancer cells. Firstly, as a result of impairment of the NRF2 pathway, cancer cells may employ different strategies to neutralize high ROS levels and in turn trigger alternative apoptotic pathways. Secondly, inhibition of NRF2 decreases cancer cells’ ability to remove excess ROS, thus sensitizing them to chemotherapeutics and reversing MDR [7].

### 4.1. NRF2 Activators

NRF2 activators enhance cells’ ability to detoxify carcinogens, mostly by reacting with KEAP1 cysteine residues, intercepting KEAP1-dependent degradation of NRF2 [20].

Curcumin, also known as diferuloylmethane, is a well-investigated polyphenol found in *Curcuma longa*, which possesses anti-inflammatory, antioxidant, and anticancer properties, and it also acts mainly as an NRF2 activator. Its mechanisms of activation consist of demethylation of the NRF2 promoter region and promotion of nuclear translocation of NRF2, and it also interferes with KEAP1 by directly interacting with its sensor cysteine thiols, subsequently preventing NRF2 degradation. Other mechanisms include stimulation of PKC which phosphorylates NRF2 or activation of upstream kinases, like MAPK. It affects the expression of various NRF2-dependent proteins which include *HO-1*, *NQO1*, GST, GR, AR, and phase-II detoxifying and antioxidant enzymes [9,21,22,23]. Curcumin has also shown to increase the sensitivity of prostate, ovarian and colorectal cancers to both radiotherapy and chemotherapy [9]. Unfortunately, curcumin exhibits poor pharmacokinetic properties, very low bioavailability, and quick metabolism, which hinder its use in therapy [22]. Moreover, curcumin may act as an NRF2 inhibitor by upregulating PTEN (phosphatase and tensin homolog deleted on chromosome ten), which results in overcoming MDR in lung cancer, or by activating KEAP1, resulting in higher NRF2 polyubiquitination, which reverses cisplatin (DDP) resistance in non-small-cell lung cancer A549/DDP cells (Table 1) [2,24,25].

Epigallocatechin-3-gallate (EGCG) is a polyphenol mainly found in green tea extract. Chemically, EGCG is the ester of epigallocatechin and gallic acid (3,4,5-trihydroxybenzoic acid) and is a type of catechin. It is considered mainly as an NRF2 activator. It induces nuclear translocation of NRF2, which heightens the expression of its target genes, mainly phase-II detoxifying enzymes, resulting in an anti-inflammatory and antioxidative effect [21,23,26]. Its code of action seems to consist of either oxidizing or modifying cysteine residues of KEAP1, stimulating disassociation of NRF2 from the complex. Other possibilities include activation of NRF2 via phosphorylation of its serine/threonine residues by ERK and PI3K or inhibition of BACH1 expression. EGCG has proven effective in protecting against colon, lung, fore-stomach, skin, and prostate cancer. Another noteworthy property of EGCG is its ability to reduce angiogenesis, metastasis, and cancer cell invasion through expression downregulation of matrix metalloproteinases and inhibition of cell adhesion [9]. It has been shown that low doses indeed activate NRF2, whereas higher doses lead to NRF2 inhibition [22]. In one study, EGCG was successfully used to overcome MDR in tamoxifen-resistant breast cancer (Table 1) [2,27]. Real-time quantitative PCR analysis revealed that EGCG suppressed the mRNA expression of *HO-1* and *NQO1* in a dose-dependent manner. EGCG at a concentration of 50 and 100 µM reduced the mRNA levels of these genes. Interestingly, NRF2 was downregulated even more than its target genes. In MCF-7/TAM cells, NRF2 mRNA level dropped more than 53% in the presence of 50 μM EGCG. In contrast, KEAP1 mRNA level was affected less than other ones, suggesting that EGCG-mediated inhibition is independent of KEAP1. In addition, EGCG reduced protein levels of nuclear NRF2 as well as NRF2 target genes, including *HO-1* and *NQO1*, in a dose-dependent manner, consistent with their mRNA expressions [27].

Sulforaphane (1-isothiocyanato-4-(methylsulfonyl)-butane) (SFN) is an isothiocyanate found in cruciferous plants like broccoli and is an activator of NRF2, characterized by its high bioavailability. It possesses antioxidant, antigenotoxicity, and chemotherapeutic effects, and it can also induce apoptosis and reduce angiogenesis [9,22]. Its mechanism of action includes upregulation of NRF2 expression and binding to KEAP1 and subsequent activation of both NRF2 and AREs, which results in elevated levels of proteins such as *NQO1*, *HO-1*, GST, and phase-II detoxification enzymes [9,28]. The effects of SFN were measured in human hepatocytes (HHL5) and hepatoma (HepG2) cells [29]. Results showed that SFN inhibited cell viability and induced DNA damage at high doses (≥20 μM). Pre-treatment with a low dose of SFN (≤5 μM) protected against hydrogen peroxide (H_2_O_2_)-induced cell damage. High doses were more toxic towards HHL5 compared to HepG2 cells. In addition, HepG2 cells captured the cytoprotective effect of SFN over a wider dose range (1.25–20 μM) compared to HHL5. Manipulation of levels of GSH and NRF2 in HepG2 cells confirmed that both molecules mediate the protective effects of SFN against H_2_O_2_. However, the nonspecific nature of SFN in regulation of cell death and survival could present undesirable risks, i.e., be more toxic to normal cells, and cause chemo-resistance in tumor cells [29].

Resveratrol (3,5,4-trihydroxystilbene), found mainly in various berries, grapes, and red wine, is a polyphenol with NRF2-activatory effect. It has anticancer, antioxidant, anti-inflammatory, and antidiabetic properties and plays a crucial role in modulating many signaling pathways including those associated with cellular growth and division, angiogenesis, invasion, metastasis, and apoptosis [9]. Treatment with resveratrol results in lowered KEAP1 protein levels and heightened NRF2, *HO-1*, and GST protein and phase-II detoxification enzyme levels; increased nuclear transition of NRF2 was also observed. Another mechanism of resveratrol-induced NRF2 upregulation includes activation of upstream kinases like MAPK (mitogen-activated protein kinase) [9,22,28]. Its influence on NRF2 seems to be dose-dependent as the effects described above were observed when low doses were applied, whereas high doses of resveratrol exhibit pro-oxidant activity [23].

Quercetin (3,3′,4′,5,7-pentahydroxyflavone) is a vegetable and fruit flavonol with NRF2-activatory effect. It activates NRF2 through its continuous phosphorylation by PKC (protein kinase C) and promotes nuclear translocation of NRF2 as well as expression of phase-II detoxifying enzymes while at the same time increasing those enzymes’ activity. Quercetin also interacts with KEAP1 directly, hindering its ability to bind to NRF2 [21,30].

Myricetin (3,5,7,3′,4′,5′-hexahydroxyflavonol), found in *Myrica nagi*, causes modifications to KEAP1 without interfering with its protein levels; this leads to inhibition of NRF2 polyubiquitination and its subsequent activation, which results in elevated levels of *HO-1* protein. At the same time, myricetin promotes the nuclear translocation of NRF2 [30,31].

Diallyl trisulfide (DATS), found in garlic oil and cruciferous vegetables, is an isothiocyanate. Treatment with this compound results in NRF2 activation, evidenced by increased *NQO1* and *HO-1* expression; simultaneously, the treatment lowered levels of the KEAP1 protein [9].

1-O-hexyl-2,3,5-trimethylhydroquinone (HTHQ) is a derivative of vitamin E, used in the treatment of hepatic cirrhosis, diabetes, and neurodegenerative diseases as well as cancers. Its activity causes increased nuclear translocation of NRF2 and increased expression of *HO-1*, leading to antioxidant and antiapoptotic effects [28].

Crocin (bis[β-D-glucopyranosyl-(1→6)-β-D-glucopyranosyl]8,8′-diapocarotene-8,8′-dioate), a carotenoid derivative from *Crocus sativus*, is used in the treatment of obesity, hypertension, and cardiovascular diseases. It was also able to upregulate NRF2′s activity, leading to higher expression of *HO-1* [28].

Kaurenoic acid (also known as kaur-16-en-19-oic acid), an NRF2-activatory tetracyclic diterpene, causes upregulation of *HO-1*, *NQO1*, and glutamate-cysteine ligase catalytic subunit (GCLC) protein expression [21].

Other NRF2 activators include oxymatrine [7], lycopene, glycyrrhizin [9,21], hesperidin and its derivatives [30], cordyceps acid, garlic oil [26], rosmarinic acid [21], astragaloside IV, pyrroloquinoline quinone, silibinin, fisetin [28], kahweol, organosulfur compounds, zerumbone, carnosol, cinnamonyl-based compounds, cafestol [9], 2-undecanone [26], and honaucin A [22].

### 4.2. NRF2 Inhibitors

Comparatively, only a few NRF2 inhibitors are known to date. Their anticancer potential is exhibited by their ability to sensitize cancer cells to chemotherapeutics by downregulating expression of detoxifying enzymes [9].

Luteolin (3′,4′,5,7-tetrahydroxyflavone), a polyphenolic flavonoid, can be found in vegetables like broccoli, pepper, parsley, and celery and is a documented NRF2 inhibitor (Table 1). It possesses antibacterial, anti-inflammatory, antioxidant, anticancer, and cytoprotective activities, and it has also been shown to suppress proliferation and cell cycle promotion as well as induce apoptosis in various cancers [9]. Its activity results in decreased NRF2 mRNA and protein levels, as well as impairment of binding of NRF2 to AREs and GSH depletion [4,20,32,33]. As a result, protein levels of *HO-1*, *NQO1*, MRP2, MDR1, and phase-II detoxifying enzymes are decreased [23,30,33,34]. Luteolin has proven useful in overcoming MDR in breast, colorectal, and lung cancer [2,9,34]. Two such examples include reversal of oxaliplatin (OX) resistance in colorectal cancer HCT116-OX and SW620-OX cell lines, which also sensitized them to cisplatin, doxorubicin, and bleomycin [4,35,36]. Luteolin also inhibited NRF2 target genes—*HO-1*, *NQO1* and *GSTα1/2*—expression and decreased GSH level in small intestinal cells of wild-type mice. This effect was not observed in NRF2^−/−^ mice [35]. Luteolin treatment decreased the protein level of NRF2 by 30–60% and *NQO1* by 15–40% in HCT116-OX cells. In SW620-OX cells, luteolin also caused a decrease in the protein level of NRF2 by 20–50% and *NQO1* by 15–40%. In vivo studies showed that luteolin treatment decreased the protein level of *NQO1* by 32.1%, *HO-1* by 34.7%, and GSTα1/2 by 70.6%. The level of GSH was reduced by 29% in the luteolin group of mice. In contrast, such distinct responses were not detected in NRF2^−/−^ mice [35]. Oral administration of luteolin, either alone or combined with intraperitoneal injection of cisplatin, greatly inhibited growth of xenograft tumors from non-small-cell lung cancer cell line A549 cells grown subcutaneously in athymic nude mice [36]. The total tumor weight was reduced by 55% in the luteolin and 47% in the cisplatin group compared with the control. Moreover, luteolin enhanced the toxic effect of cisplatin and the combined treatment resulted in a 70% reduction in tumor weight compared with the control. Cell proliferation, expression of NRF2, and antioxidant enzymes were all reduced in tumor xenograft tissues. The percentage of Ki67-positive cells was 41% in the control group, 31% in the luteolin group, 32% in the cisplatin group, and 25% in the combination group. Immunoblotting of tumor xenografts indicated that NRF2 expression was reduced to 60%, *NQO1* to 72%, AKR1C to 55%, and *HO-1* to 58% in the luteolin and combination treatments, whereas there was no significant reduction in the cisplatin group. Thus, luteolin enhanced the anticancer effect of cisplatin. Together, these findings demonstrated that luteolin inhibits the NRF2 pathway in vivo and can serve as an adjuvant in the chemotherapy of NSCLC [36].

Luteolin has also increased the sensitivity of siGFP-C5 (a stable lung cancer cell line developed from A549 cells by transfecting pRS-GFP) and A549 cell lines to bleomycin, oxaliplatin, and doxorubicin [9,30,32]. At the same time, however, there were instances of luteolin acting as an NRF2 activator, inducing the NRF2/ARE pathway and causing higher expression of said pathways’ downstream genes; for example, in human liver cancer HepG2 cells, increased mRNA and protein levels of NRF2 and *HO-1* were observed [9,30,32]. It was also shown that luteolin treatment induced NRF2/ARE/*HO-1* activation in HCT116 human colorectal cancer cells [37]. Luteolin can enhance the chemotherapeutic effect of oxaliplatin in HCT116 cells, potentially by shifting oxaliplatin-induced cell cycle arrest to apoptosis. In addition, luteolin and oxaliplatin caused apoptosis and cell cycle arrest, respectively, in a p53-dependent manner. Moreover, luteolin-induced NRF2/ARE/*HO-1* activation negatively regulated oxaliplatin-promoted p53 signal transduction, and oxaliplatin-induced cell cycle arrest was seemingly disturbed by luteolin-induced *HO-1* upregulation. These results suggest that luteolin could strengthen the anticancer activity of oxaliplatin in HCT116 cells in a p53-dependent manner [37].

Apigenin (4′,5,7-trihydroxyflavone) is a bioflavonoid exhibiting an inhibitory effect on NRF2 that can be found in vegetables and various beverages made from plants. It possesses antioxidant, anti-inflammatory, anticancer, and antiviral properties [9]. Apigenin’s activity reduces NRF2 at both the mRNA and protein level through downregulation of the PI3K/Akt pathway and hinders its nuclear translocation [19,26,30]. Other effects also include reduced mRNA and protein levels of phase-II detoxifying enzymes [26,30]. The inhibitory effect of apigenin was demonstrated in BEL-7402 cells (human hepatocellular carcinoma), which had their NRF2 expression lowered [9]. Apigenin was also able to sensitize BEL-7402/ADM cells to doxorubicin (also known as Adriamycin (ADM)) (Table 1) [19,30,38,39]. Apigenin-mediated sensitization to doxorubicin relies on its ability to suppress the NRF2 pathway through reducing the nuclear NRF2 protein level as well as hindering xenobiotic metabolism and the activity of antioxidant enzymes such as *HO-1*, AKR1B10, a human member of the aldo-keto reductase superfamily, and MRP5 (multidrug resistance-associated protein 5) in BEL-7402/ADM cells [38]. Real-time quantitative PCR analysis revealed that apigenin (20 µM) suppressed the mRNA expression of *HO-1*, *AKR1B10*, and *MRP5* genes by 27, 16, and 30%, respectively. In BEL-7402/ADM cells, NRF2 mRNA level decreased more than 39%. In contrast, KEAP1 mRNA level was not affected, suggesting that apigenin-mediated inhibition is independent of KEAP1. In addition, apigenin reduced protein levels of nuclear NRF2 as well as NRF2 target genes, including *HO-1*, *AKR1B10*, and *MRP5*, consistent with their mRNA expressions. Moreover, apigenin and doxorubicin co-treatment inhibited tumor growth, reduced cell proliferation, and induced apoptosis more substantially when compared with doxorubicin treatment alone in BEL-7402 xenografts. Apigenin significantly reversed doxorubicin resistance and induced caspase-dependent apoptosis in BEL-7402/ADM cells [39]. Apigenin induced miR-101 expression, and overexpression of miR-101 mimicked the doxorubicin-sensitizing effect of apigenin. Importantly, it was shown that miR-101 was able to target the 3′-UTR of NRF2. The results of these studies suggested that apigenin sensitizes BEL-7402/ADM cells to doxorubicin through the miR-101/NRF2 pathway [39]. At the same time, there are examples of apigenin acting as an NRF2 activator. It may increase NRF2 transcription and, as a result, elevate protein levels of phase-II detoxification enzymes. Such effect was exemplified in a t-BHP-treated retinal pigment epithelia (RPE) cell line (ARPE cells), in which heightened NRF2 and *HO-1* mRNA expression was observed. Apigenin was also able to restore silenced NRF2 in the skin epidermal JB6 P + cell line [9]. Another issue with apigenin is its low solubility and absorption, which makes use of carriers necessary [26].

Berberine (2,3-methylenedioxy-9, 10-dimen thoxyprotoberberinechloride) is an isoquinoline alkaloid found in various plants from the *Berberis* family as well as in *Coptis chinensis* and *Hydratis canadensis* (Table 1) [18,40]. It has a wide range of bioactivities, exhibiting, among others, antiobesity, cholesterol-lowering, antimicrobial, hepatoprotective, anti-inflammatory, and antioxidant effects, which make this compound a promising object for use in neurodegenerative, metabolic, and cardiovascular diseases, as well as in cancer prevention, treatment, and drug sensitization [40,41,42]. It is characterized by low bioavailability caused by its low absorption and fast metabolism, and thus its chloride and sulfate salts are used [42]. Berberine exhibits a dual effect on cell survival, as it seemingly promotes death of cancer cells while at the same time protecting normal cells, to which it exhibits comparatively much lower toxicity [7]. Its anticancer activity stems from its anti-inflammatory and proapoptotic activity, its ability to induce cell cycle arrest, reduce expression of proteins responsible for metastasis, and inhibit telomerase activity, as well as its ability to sensitize MDR cancers to chemotherapeutics [7,43,44]. Berberine garnered interest as it has been shown to sensitize various types of cancer to drugs such as doxorubicin, cisplatin, 5-fluorouracil (5-FU), niraparib, icotinib, and osimertinib [41,42]. Berberine also proves to be useful in sensitizing cancers to radiotherapy, with clear evidence pointing to the involvement of NRF2 in this process [18,43].

Lapatinib, a tyrosine kinase inhibitor of HER2/EGFR, is used to treat HER2-positive breast cancer [45]. It was found that a new combination therapy of berberine with lapatinib overcame lapatinib resistance. Lapatinib activated both the c-Myc/pro-NRF2 pathway and GSK-3b signaling to stabilize NRF2 and maintain a low level of ROS in resistant BT-474^LapR^ and AU-565^LapR^ cells. Berberine induced apoptosis of lapatinib-resistant cells through ROS level’s upregulation. Therefore, berberine was able to sensitize breast cancer cell lines to lapatinib by inhibiting the NRF2 pathway and causing higher accumulation of ROS. These finding provide a novel strategy of using berberine to overcome lapatinib resistance [45].

Mechanisms by which berberine modulates NRF2 activity include the suppression of KEAP1, influencing NRF2 nuclear translocation, changing NRF2 mRNA expression, and affecting upstream mediators of NRF2 [42]. Berberine may cause both inhibitory and activatory effects on NRF2, but unfortunately, as of now, it is not exactly known what factors contribute to either outcome [40,46].

Trigonelline (1-methylpyridin-1-ium-3-carboxylate) is an alkaloid with NRF2-inhibitory properties (Table 1). It can be found in coffee amongst many other plants. Trigonelline possesses anticancer, hypocholesterolemic, antimigraine, and antidiabetic properties. Its main code of actions seems to be blockage of nuclear translocation of NRF2, resulting in lower expression of its target genes [9,22]. This has been exemplified in a study where pretreatment of non-small-cell lung cancer (NSCLC) A549 and NCIH460 cell lines with trigonelline caused lower expression of NRF2-dependent genes such as *HO-1* and *NQO1*, while the level of cellular NRF2 remained unchanged [7,47]. It was shown that mRNA levels of *NQO1* and *HO-1* declined by more than 5-fold in both A549 and NCIH460 cells in the presence of 50 µM trigonelline. Trigonelline also prevented nuclear accumulation of pNRF2 (4-fold decrease). Moreover, it was shown that trigonelline inhibits NRF2 activation and cell proliferation via blocking the activation of the epidermal growth factor receptor (EGFR) signaling pathway [47]. In other works, trigonelline has been shown to reverse ferroptosis resistance in NRF2-activated head and neck cancers [9]. Its ability to reverse resistance to oxaliplatin in colon cancer SW480 cells has also been proven [48]. Applying NRF2 inhibition by trigonelline (500 nM), trigonelline-loaded 3Block (PCL-PEG-PCL) (500 nM) and trigonelline-loaded 5Block (PLA-PCL-PEG-PCL PLA) (500 nM) micelles caused an almost 2-fold decrease in NRF2 mRNA expression in resistant colon cancer cells. In addition, *HO-1* mRNA expression revealed a 3-fold decrease after applying trigonelline and trigonelline-loaded 3Block micelles. The results also showed a 10-fold decrease in *HO-1* mRNA expression when the cells were incubated with trigonelline-loaded 5Block micelles for 24 h, suggesting that although trigonelline-loaded 5Block micelles had almost the same impact on NRF2 suppression, the inhibition efficiency induced by this nanoparticle was expressed more significantly in downstream genes including *HO-1* and *NQO1* [48].

Brusatol found in *Brucea javanica* is a quassinoid exhibiting NRF2-inhibitory effects [49]. It enhances NRF2 polyubiquitination without affecting KEAP1 protein level, leading to the decreased protein level of NRF2 and suppression of its target genes, like *HO-1* and *NQO1* [6,9,20,32,50]. Subsequent disruption of antioxidant defense has proven to enhance the cytotoxic effect of anticancer drugs such as cisplatin, 5-fluorouracil, carboplatin, etoposide, and paclitaxel. It was shown that co-treatment with brusatol and cisplatin in lung cancer in the xenograft model resulted in improved efficacy of therapy [6,9,20,33]. Other satisfactory results of brusatol use include sensitizing HeLa (cervical cancer), MDA-MB-231 (breast cancer), and A549 (non-small-cell lung cancer) cells to chemotherapeutics [9]. However, it is apparent that brusatol is not NRF2-specific and acts as a global protein synthesis suppressor, which limits its potential use [20,22,32].

Recently, it was also found that brusatol sensitized endometrial cancer to progestin by suppressing NRF2-TET1 AKR1C1-mediated progestin metabolism [51]. *AKR1C1* is well characterized as a target gene of NRF2. Increased AKR1C1 expression might be associated with the pathological progression of endometrial cancer. Brusatol transcriptionally suppressed *AKR1C1* via modifying the hydroxymethylation status in its promoter region through TET1 inhibition. Suppression of AKR1C1 by brusatol resulted in decreased progesterone catabolism and maintained potent progesterone to inhibit endometrial cancer growth. This inhibition pattern has also been found in the established xenograft mouse and organoid models [51].

The combination of Ara-C (cytarabine) and brusatol synergistically exerted a significant proapoptotic effect in human acute myeloid leukemia (AML) cells, HL-60, and THP-1 [52]. The synergistic antitumor effect of Ara-C/brusatol in AML cells was mediated by attenuating NRF2 expression. Moreover, NRF2 inhibition by brusatol caused downregulation of the expression of glycolysis-related proteins and decreased glucose consumption and lactate production, whereas the level of ROS production was unaffected. The activation of NRF2 by sulforaphane could reverse the chemotherapeutic effect and cause changes in glycolysis upon concomitant treatment with Ara-C and brusatol in AML cells. Ara-C/brusatol co-treatment decreased glucose-6-phosphate dehydrogenase (G6PD) protein expression and increased the sensitivity of Ara-C. In vivo experiments with a mouse xenograft confirmed that combining Ara-C with brusatol resulted in a stronger antileukemia effect than Ara-C alone [52].

Brusatol exerted growth-inhibitory effects on HER2-positive cancer cells by regressing NRF2/*HO-1* and HER2-AKT/ERK1/2 signaling pathways in these cells [53]. Interestingly, brusatol synergistically enhanced the antitumor activity of trastuzumab against HER2-positive SK-OV-3 and BT-474 cells, which may be attributed to the inhibition of NRF2/*HO-1* and HER2-AKT/ERK1/2 signaling pathways. Furthermore, synergistic effects were also observed in BT-474 and SK-OV-3 tumor xenografts. In addition, it was shown that trastuzumab markedly enhanced brusatol-induced ROS accumulation and apoptosis level, which could further explain the synergistic effect. This study provided a new insight into NRF2 inhibition in combination with HER2-targeted trastuzumab as a potential clinical treatment regimen for treating HER2-positive cancers [53].

Moreover, brusatol effectively enhanced the antitumor effect of lapatinib against HER2-positive SK-BR-3, SK-OV-3, and AU565 cancer cells in a synergistic manner [54]. The combination of lapatinib with brusatol decreased NRF2 levels and induced ROS generation in both SK-BR-3 and SK-OV-3 cells more effectively than lapatinib alone. A significant reduction in the phosphorylation of HER2, EGFR, AKT, and ERK1/2 in SK-BR-3 and SK-OV-3 cells was also observed when treated with lapatinib and brusatol compared to either agent alone. More importantly, brusatol significantly augmented the antitumor effects of lapatinib in the SK-OV-3 xenograft model. These data indicate the potential for the use of a combination of brusatol and lapatinib in HER2-positive cancer treatment [54].

Chrysin (5,7-dihydroxyflavone) is a flavonoid with NRF2-inhibitory effect found in *Passiflora caerulea* as well as various fruits and vegetables. It has anticancer, antioxidant, anti-inflammatory, antidiabetic, and antiaging effects, as well as hepato- and neuroprotective properties. It has also been widely proven that chrysin plays a pivotal role in the suppression of proinflammatory cytokine expression, downregulation of NF-kB, TNF-α, and IL-1β, upregulation of apoptotic pathways, and prevention of angiogenesis and metastasis formation [9]. Its activity results in lowering of mRNA and protein levels of NRF2 by downregulating ERK and PI3K/Akt signaling pathways as well as disruption of its nuclear translocation [4,22,34]. In turn, expression of NRF2-dependent genes like *HO-1*, *NQO1*, *AKR1B10, MRP1,* and *MRP5* is decreased, as exemplified in breast cancer MCF-7 cells, which have become more susceptible to doxorubicin after chrysin treatment [4,9,30]. Another effect of chrysin administration was downregulation of phase-II detoxifying enzymes in doxorubicin-resistant hepatocellular carcinoma BEL-7402/ADM cells and their sensitization to chemotherapy [4,30,55]. The mRNA levels of *HO-1*, *AKR1B10,* and *MRP5* genes were reduced 42%, 39%, and 45% by 20 µM of chrysin, respectively. In BEL-7402/ADM cells, the NRF2 mRNA level dropped more than 60% in the presence of 20 µM chrysin (Table 1). Moreover, it was shown that chrysin increased (35%) intracellular accumulation of doxorubicin compared to the untreated cells [55]. It was also shown that chrysin can hinder proliferation, migration, and invasion of human T98, U251, and U87 glioblastoma (GBM) cells and abrogate the in vivo tumorigenicity of U87 xenografts in BALB/c mice [22].

All-trans retinoic acid or ATRA acts as an NRF2 inhibitor through directly binding to it through retinoic receptor alpha (RARα) and blocking its transactivation abilities, regardless of NRF2’s subcellular location [6,19,56]. It is, however, worth noting that ATRA’s activity is not NRF2-specific [6].

Wogonin (5,7-dihydroxy-8-methoxyflavone) is another flavonoid with NRF2-inhibitory activity than can be found in *Scutellaria baicalensis Georgi*. It causes suppression of the NRF2/ARE pathway and reduces protein levels of downstream genes of NRF2 such as *HO-1*, *NQO1*, and *MRP1*. One of possible ways wogonin causes NRF2 inhibition seems to be through hindering of NF-κB binding to the NRF2 promoter [4,22,23]. Wogonin treatment was able to reverse cisplatin and doxorubicin resistance in various cell lines such as head and neck cancer (HNC) cells and breast cancer MCF-7 cells (Table 1) [4,57,58]. Kim et al. demonstrated that wogonin could act synergistically with cisplatin and thereby circumvent the resistance to cisplatin in HNC cells [58]. Cisplatin is a first-line chemotherapeutic agent for HNC. The combination of cisplatin and wogonin may be effective in a clinical setting to reduce cisplatin toxicity and overcome anticancer drug resistance. Wogonin induced a strong increase in cisplatin-mediated apoptosis via BAX, PUMA, and PARP cleavage in cisplatin-resistant HNC cells. Interestingly, wogonin showed a preventative effect on weight loss and blood cell count changes in mice treated with cisplatin. Taken together, these findings may be of paramount clinical significance as the induction of cell death in cisplatin-resistant cells by wogonin could reduce the dose of cisplatin required in a clinical setting and thereby minimize the potential adverse effects of this drug. In conclusion, current data suggest that wogonin selectively kills HNC cells by inducing intracellular ROS accumulation with no notable cytotoxic effects against normal cells. A novel mechanism by which wogonin can trigger HNC cell death via JNK and PARP activation resulting from NRF2-GSTP1 inhibition was also revealed. Therefore, wogonin can reverse cisplatin resistance in HNC cells, which is associated with enhanced cellular oxidative stress via NRF2 [58].

It was observed that wogonin reduced NRF2 nuclear translocation and therefore elevated the level of intracellular ROS for the purpose of killing malignant cells. Furthermore, the suppression of NRF2 by wogonin can potentiate cytotoxic effects of chemotherapeutic agents such as hydroxycamptothecin, cisplatin, and etoposide in HepG2 cells [59]. On one hand, downregulation of NRF2 leads to a reduction in cytoprotective effect by inducing phase-II enzymes, which sensitize cells to chemotherapeutic agents. On the other hand, inhibition of multidrug resistance-associated proteins (MRPs) by wogonin enhances the effective drug level in cancer cells and potentiates their chemotherapeutic effects. It was also found that the decrease in NRF2 levels may be related to overexpression of p53. Using p53 siRNA to knock down the endogenous p53 expression, the levels of both c-Myc and NRF2 in the nucleus increased when exposed to wogonin. These results indicate that wogonin can be used in chemotherapy not only because of its own antitumor activity but also due to its ability to enhance the cytotoxic effects of chemotherapeutic agents [59].

Wogonin had strong reversal potency by inhibiting functional activity and expression of MRP1 in both proteins and mRNA in doxorubicin-induced resistant human myelogenous leukemia K562/A02 cells [60]. Wogonin-mediated inhibition of the NRF2/ARE signaling pathway in K562/A02 cells occurred through the reduction in NRF2 mRNA at the transcriptional level rather than RNA degradation. Wogonin-mediated activity of the NRF2/ARE pathway is regulated primarily through post-transcriptional regulation. Other findings also showed a strong correlation between downregulation of the PI3K/Akt survival pathway and NRF2/ARE inhibition as a result of the wogonin-induced reversal effect in K562/A02 cells, and this was correlated with diminished DNA-dependent protein kinase catalytic subunit (DNA-PKcs) level, which is an enzyme belonging to the PI3K superfamily. These results indicate a possible mechanism by which inhibition of NRF2/ARE activity plays a role in the wogonin-induced downregulation of MRP1, resulting in a reversal effect in the doxorubicin-induced resistant human myelogenous leukemia cells [60].

Ginkgetin is the first known biflavonoid, isolated from the leaves of *Ginkgo biloba* L. after which it was named. It is a derivative of amentoflavone with two methoxy groups (7,4′-dimethylamentoflavone). Ginkgetin exhibits suppressive activity on NRF2. It also promotes autophagy and causes inhibition of *HO-1* expression, leading to high ROS levels, exemplified by its synergic cytotoxic effect with cisplatin when used on NSCLC cells [26,31]. Ginkgetin synergized with cisplatin to increase cytotoxicity in NSCLC cells, which was concomitant with an increased labile iron pool and lipid peroxidation [61]. Both of these processes are key characteristics of ferroptosis. The induction of ferroptosis mediated by ginkgetin was further confirmed by lowered expression of SLC7A11 and GPX4, and a decreased GSH/GSSG ratio. Simultaneously, ginkgetin disrupted redox hemostasis in cisplatin-treated cells, as demonstrated by the enhanced ROS formation and inactivation of the NRF2/*HO-1* axis. Ginkgetin also enhanced cisplatin-induced mitochondrial membrane potential (MMP) loss and apoptosis in cultured NSCLC cells [61].

Febrifugine (3-{3-[(2*R*,3*S*)-3-hydroxypiperidin-2-yl]-2-oxopropyl}quinazolin-4(3*H*)-one) extracted from *Dichroa febrifuga* is an alkaloid NRF2 inhibitor. Its derivative—halofuginone—was able to suppress accumulation of NRF2 in cancer cells which had previously displayed high levels of this protein [22,50]. Halofuginone’s mechanism of NRF2 downregulation is caused by inhibition of prolyl-tRNA synthetase; this has led to lower expression of enzymes crucial for GSH and iron metabolism, ROS scavenging, and drug transport and metabolism. It has also proven its ability to impair proliferation, migration, and invasion of cancer cells as well as reverse radioresistance and improve drug delivery [20,22]. Halofuginone is a less-toxic febrifugine derivative that rapidly suppresses the accumulation of NRF2 protein in NRF2-addicted cancer cells. It was found that halofuginone treatment enhanced the sensitivity of NRF2-addicted cancer cells to anticancer drugs both in vitro and in vivo. These results unequivocally demonstrate that halofuginone serves as a chemosensitizer by inhibiting NRF2 accumulation in NRF2-addicted cancer cells [62].

Halofuginone has been shown to inhibit tumor progression in a variety of animal models such as bladder carcinoma, glioma, hepatocellular carcinoma, and prostate cancer [63]. Recently, it was demonstrated that halofuginone strengthened 5-aminolevulinic acid photodynamic therapy (ALA-PDT) inhibition of human epidermoid carcinoma (SCL-1) cell viability and migration, as well as NRF2-related β-catenin, p-Erk1/2, p-Akt, and p-S6K1 expression. Overexpression of NRF2 conferred resistance to the co-treatment’s effects on c-Myc, Cyclin D1, and Bcl-2, as well as cell proliferation. Halofuginone also strengthened ALA-PDT’s inhibition of tumor volume in a cutaneous squamous cell carcinoma (cSCC) mouse model and elevated ROS generation of ALA-PDT [63].

2′,4′-dihydroxy-6′-methoxy-3′,5′-dimethylchalcone (DMC) isolated from *Cleistocalyx operculatus* is yet another NRF2 inhibitor (Table 1) [4,64]. Treatment with DMC caused decreased expression of NRF2 and blocked its nuclear translocation as well as its binding to AREs, resulting in decreased levels of GSH in human hepatocellular carcinoma BEL-7402/5-FU cells [4,65,66]. Administration of DMC reversed the multidrug resistance of BEL-7402/5-FU cells significantly. DMC enhanced the sensitivity of BEL-7402/5-FU cells not only to 5-fluorouracil (5-FU) but also to doxorubicin. Staining with Hoechst 33258 and flow cytometric analysis showed that DMC has an apoptosis-inducing effect on BEL-7402/5-FU cells. It was also observed that overexpression of the multidrug resistance-associated protein (MRP1) and of glutathione S-transferase π (GST-π) contributed to MDR in BEL-7402/5-FU cells. The mRNA expressions of MRP1 and GST-π and the protein expression of MRP1 were decreased by DMC. These data demonstrated that DMC could effectively reverse MDR in BEL-7402/5-FU cells [65]. DMC treatment for 48 h at 4, 8, and 16 µM, declined the intracellular GSH content of BEL-7402/5-FU by 8%, 20%, and 51%, respectively. The GST activity was significantly higher in BEL-7402/5-FU cells compared with that in BEL-7402 cells. DMC at 4 µM reduced the GST activity by 16%, and 16 µM DMC reduced it by 55%. DMC could completely suppress the increased GST activity in BEL-7402/5-FU cells in a dose-dependent manner. It was also shown that the ADP/ATP ratio in BEL-7402/5-FU cells significantly increased after incubation with DMC. All the results above suggested that DMC might contribute to decreased drug efflux by downregulating the level of GSH and ATP as well as the GST activity in BEL-7402/5-FU cells. Moreover, the total NRF2 protein level was significantly higher in BEL-7402/5-FU cells compared with that of the BEL-7402 cells. When treated with 10 µM DMC for 4 h and 8 h, the total NRF2 protein level was decreased by approximately 87.5% and 97%, respectively. Thus, DMC affected the expression of NRF2 in BEL-7402/5-FU cells [66]. DMC was found to trigger apoptosis predominantly via the mitochondria-dependent pathway and the enhancement of reactive oxygen species (ROS) generation [67]. DMC induced G1 cell cycle arrest through downregulation of cyclin D1 and CDK4. Furthermore, DMC increased p53 level and inhibited NF-κB nuclear localization via suppression of the PI3K/AKT signaling axis, which might be the underlying mechanism of DMC-induced apoptosis and cell cycle arrest in BEL-7402/5-FU cells. Collectively, the study elucidated the mechanisms by which DMC may inhibit the growth of BEL-7402/5-FU cells and suggested the possibility that DMC might be a promising candidate therapeutic agent for hepatoma treatment in the future [67].

3′,4′,5′,5,7-pentamethoxyfavone (PMF) is a flavonoid compound found in the *Rutaceae* family, which has been shown to reduce expression of NRF2 and its downstream genes—*HO-1*, *NQO1*, and *GCLC*. Treatment of A549/CDDP cells with 3′,4′,5′,5,7-pentamethoxyfavone has led to sensitization to cisplatin (Table 1) [4,34,68]. Expression levels of NRF2 and its target genes *GCLC*, *HO-1*, and *NQO1* were significantly higher in cisplatin-resistant A549 (A549/CDDP) cells than those in A549 cells, and this resistance was partially reversed by NRF2 siRNA [68]. PMF sensitized A549/CDDP to cisplatin and substantially induced apoptosis compared with that of the group treated with cisplatin alone, and this reversal effect decreased when NRF2 was downregulated by siRNA. These results demonstrated that PMF could be used as an effective adjuvant sensitizer to increase the efficacy of chemotherapeutic drugs by downregulating the NRF2 signaling pathway [68].

Extracts from *Castanea crenata*’s (chestnut) leaves have also presented an NRF2-inhibitory effect [34]. It was found that MCF-7-derived cancer stem cells (CSCs) with a CD44^high^/CD24^low^ phenotype formed mammospheres and highly expressed NRF2 and exhibited less sensitivity to paclitaxel compared to adherent parental breast cancer MCF-7 cells [69]. *Castanea crenata* (chestnut) leaf extract significantly decreased the nuclear translocation of NRF2 and protein expression of antioxidant enzymes in MCF-7-derived CSCs. The combined treatment of the CSCs with chestnut leaf extract and paclitaxel resulted in more effective cell death than the treatment with paclitaxel alone (Table 1) [69]. It was observed that co-treatment with the extract at 50 µg/mL and paclitaxel at ≥1 nM significantly reduced the viability of CSCs, compared to the treatment with paclitaxel alone but did not have any significant effect on the viability of MCF-7. Co-treatment with the extract and paclitaxel decreased the expression ratio of Bcl-2 to Bax and increased the level of cytochrome C and cleaved PARP compared to the untreated cells or cells treated with paclitaxel alone. Chestnut leaf extract also facilitated paclitaxel-induced mitochondrial damage, a hallmark of apoptotic cell death. Moreover, it was shown that chestnut leaf extract hindered colony formation of CSCs and that combination of the extract and paclitaxel inhibited clonal expansion of CSCs more effectively than either treatment alone. These interesting results indicated that the extract of chestnut leaf inhibited the function of NRF2, as a master regulator of cellular antioxidant responses, and attenuated paclitaxel resistance of the CSCs [69].

**Table 1:** Effective NRF2 inhibitors in overcoming MDR in cancer.

Name	Type of Compound	Chemical Structure	Cancer Cell Line	Drug	Mechanism of Action	Ref.
Curcumin *	Polyphenol	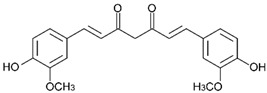	A549/DDP	Cisplatin	PTEN protein ↑ KEAP1 protein ↑	[25]
EGCG *	Polyphenol	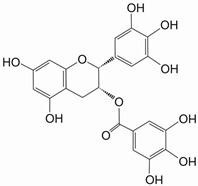	MCF-7/TAM	Tamoxifen	NRF2 mRNA ↓ NRF2 protein ↓	[27]
Luteolin **	Flavonoid	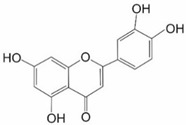	HCT 116-OX, SW620-OX	Oxaliplatin	NRF2 protein ↓	[35]
Apigenin **	Bioflavonoid	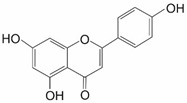	BEL-7402/ADM	Doxorubicin	PI3K/Akt/NRF2 ↓ miR-101/NRF2 ↓	[38] [39]
Berberine **	Alkaloid	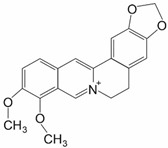	BT-474-Lap^R^ AU-565-Lap^R^	Lapatinib	NRF2 protein ↓	[45]
Trigonelline	Alkaloid	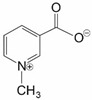	SW480/Res	Oxaliplatin	NRF2 mRNA ↓	[48]
Chrysin	Flavonoid	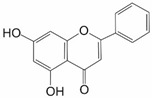	BEL-7402/ADM	Doxorubicin	PI3K/Akt/NRF2 ↓ ERK/NRF2 ↓	[55]
Wogonin	Flavonoid	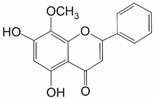	MCF-7/DOX	Doxorubicin		[57]
			AMC-HN4-cisR AMC-HN9-cisR	Cisplatin	NRF2 pathway ↓	[58]
			K562/A02	Doxorubicin	NRF2 mRNA ↓	[60]
2′,4′-dihydroxy-6′-methoxy-3′,5′-dimethylchalcone (DMC)	Chalcone	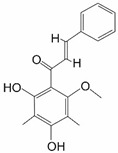	BEL-7402/5-FU	5-FU	NRF2 protein ↓	[65,66,67]
3′,4′,5′,5,7-pentamethoxy flavone (PMF)	Flavonoid	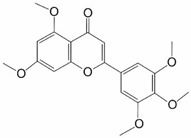	A549/CDDP	Cisplatin	NRF2 protein ↓	[68]
Extract from *Castanea crenata’s* (chestnut) leaves	-	-	MCF-7-derived CSCs	Paclitaxel	NRF2 pathway ↓	[69]

* considered mainly as activators; ** may have an activatory effect; ↑—increase; ↓—decrease.

Camptothecin ((*S*)-4-ethyl-4-hydroxy-1*H*-pyrano [3′,4′:6,7]indolizino [1,2-b]quinoline-3,14-(4*H*,12*H*)-dione) is a known topoisomerase inhibitor which was isolated from the stem wood of *Camptotheca acuminata* [70]. Camptothecin has also exhibited NRF2-inhibitory activity, suppressing its expression and transcriptional activity. Camptothecin sensitized HepG2, SMMC-7721, and A549 cell lines to epirubicin and 5-FU [4].

Oridonin (also known as isodonol or rubescenin A), a diterpenoid isolated from *Rabdosia rubescens*, prevents nuclear translocation of NRF2 and as a result lowers expression of *NQO1* and *HO-1*, leading to ROS-induced apoptosis [22].

Hederagenin (3β,23-dihydroxyolean-12-en-28-oic acid) is a triterpenoid which can be found in *Hedera helix* L. Its inhibitory effect on the NRF2/ARE pathway selectively induced death in both resistant and nonresistant HNC cells by depleting GSH and increasing ROS levels [4,71].

Isoliquiritigenin (2′,4,4′-trihydroxychalcone) isolated from *Glycyrrhiza uralensis* is a phenolic compound which was able to inhibit NRF2 activity by upregulating KEAP1 transcription and expression, resulting in the promotion of NRF2 degradation in human liver cancer HepG2 cells and leading to lowered levels of NRF2-dependent antioxidants [4,72].

Cinnamomi cortex extract (CCE) inhibits NRF2 by blocking its nuclear translocation and mRNA expression, leading to a lowered level of *NQO1* activity. It has also caused increased NRF2 degradation in lung cancer A549 cells and sensitized them to chemotherapy [34,73,74]. Moreover, it was demonstrated that CCE significantly enhances the sensitivity of A549 cells to the cytotoxic action of doxorubicin and etoposide as well as increasing the intracellular accumulation of both drugs. Bioactivity-guided fractionation revealed that procyanidin tetramers and pentamers contained in CCE were active components in suppressing NRF2 [73]. Next, it was shown that CCE procyanidin treatment rapidly reduced nuclear NRF2 expression and phosphorylated insulin-like growth factor-1 receptor (IGF-1R) in A549 cells. NRF2 suppression by CCE procyanidins was eased in the presence of protease inhibitors, not proteasome inhibitors. In addition, CCE procyanidin treatment led to the enhancement of nuclear cysteine protease activity in A549 cells. These findings suggest a novel, interesting mechanism by which CCE procyanidins can promote proteasome-independent degradation of nuclear NRF2 through IGF-1R phosphorylation and cysteine protease activation [74].

Other NRF2 inhibitors include cordycepin, cardamonin, ginsenoside Rd, lyngbyabellin A, grassypeptolide A, dolastatin 12, *Pseudomonas aeruginosa* mannose-sensitive hemagglutinin (PA-MSHA), altersolanol B [33], convallatoxin, parthenolide [7], tiliroside, withaferin A [31], plumbagin [22], and cryptotanshinone [4].

## 5. Limitations and Future Research

Compounds of plant origin exhibit a wealth of biological activities and are used as drugs in many human diseases, including cancer. They are usually more cytotoxic to cancer cells and show moderate cytotoxicity to normal cells. For example, EGCG is shown to be significantly more cytotoxic towards cancer cells rather than normal cells with initial toxicity concentrations ranging from 25 μM to 100 μM for cancers and from 150 μM to 200 μM for normal cells from the human oral cavity after 72 h exposure [75]. 2′,4′-dihydroxy-6′-methoxy-3′,5-dimethylchalcone (DMC) exhibited significant cytotoxic potential with an IC_50_ value of 32.3 ± 1.13 μM against SMMC 7721 cells (human hepatoma). DMC was shown to be slightly toxic towards normal cells from L-02 (human normal liver) and HFL-1 (human fetal lung fibroblast) lines with IC_50_ values around 111 μM and 152 μM, respectively [76]. Luteolin has shown slight toxicity towards human lung fibroblasts (TIG-1) with an IC_50_ of 107 μM, and high toxicity for human umbilical vein endothelial (HUVE) cells with an IC_50_ of 57 μM [77]. Quercetin was almost nontoxic for TIG-1 cells and highly toxic for HUVE cells with an IC_50_ of 303 μM and 61 μM, respectively [77]. Despite cytotoxicity data against normal cells being rather limited, it was noted that curcumin has a cytotoxic effect on murine macrophages and kidney cells at relatively small concentrations (IC_50_ equal to 31 μM and 15.2 μM, respectively) [78].

However, it seems that a much bigger problem in the therapeutic use of natural compounds is their low bioavailability, poor water solubility, rapid metabolism, and chemical instability rather than their potential cytotoxicity towards normal cells. Therefore, many studies are currently focused on improving the pharmacokinetic properties of substances isolated from plants. One of the strategies for increasing curcumin’s bioavailability, solubility, and its other properties includes the use of nanocurcumin which is obtained by encapsulating it into nanoformulations through ionic gelation and antisolvent precipitation, among many other methods [79]. In addition to nanoformulation, another approach to improving the pharmacological parameters of curcumin is through chemical modifications. Many findings have indicated to the diketo moiety as the site for instability of curcumin and have postulated that the incorporation of a heterocyclic ring structure in the center of the compound scaffold would not only improve the stability of the molecule but would also improve its biological activity [80]. Other strategies of curcumin delivery include the use of various biopolymer nanoparticles (human serum albumin, zein-based, silk-based, chitosan, alginate, starch, cellulose), exosomes, co-polymers, and targeted delivery [81]. Luteolin’s restricted clinical applicability caused by its low water solubility and bioavailability from food can be overcome with the use of nanoparticles (Poly(propylene sulfide)-poly(ethylene glycol) (PPS-PEG), folic acid-modified poly(ethylene glycol)-poly(ecaprolactone) (Fa-PEG-PCL) nano-micelles) and nano-drug delivery systems (NDDSs), like double-emulsion solvent volatilization or titration and single-emulsion solvent volatilization methods [82,83]. Apigenin’s applicability is hindered by its low water solubility and bioavailability. Several drug delivery systems have been applied to surmount this issue, such as liposomes, polymeric micelles, solid dispersion, carbon nanopowders, self-micro-emulsifying drug delivery systems, nanosuspensions, and nanocrystals, as well as nanostructured lipid carriers, emulsions (including micro- and nano-emulsions), hydrogels, and nanoparticles (mesoporous silica nanoparticles (MSNs), nanostructured lipid carriers (NLCs)) [84,85]. Trigonelline-loaded water-soluble chitosan nanoparticles (Trigo-WSCS NPs) were prepared for the treatment of glioblastoma (targeting C6 glioma cells) and had their biocompatibility evaluated with rat adrenal pheochromocytoma cells (PC12 cells) [86]. Trigo-WSCS NPs were noted to have a spherical shape, with an average size of 356 nm. Trigo-WSCS NPs’ zeta potential was 30.9 mv, which expresses their good stability. The WSCS-Trigo NPs considerably inhibited the growth of rat C6 glioma cells and exhibited an IC_50_ concentration of 34 μg/mL. Furthermore, Trigo-WSCS NPs were biocompatible with PC12 cells to enhance neurite growth and differentiation [86]. It was also demonstrated that trigonelline overcomes oxaliplatin resistance in colon cancer SW480/Res cells with greater impact when loaded in micelles [48]. Applying trigonelline-loaded 5Block micelles strongly increased the effect of trigonelline in promoting oxaliplatin-induced apoptosis. In the cells treated with trigonelline-loaded 5Block micelles, oxaliplatin-induced apoptosis markedly increased up to 37.1% (25.9% early, 11.2% late) compared to 15.59% (9.07% early, 6.52% late) for oxaliplatin only [48].

Chrysin’s low bioavailability and solubility can be combated with micronization, nanosuspension, derivatization, or complexation. In one study, complexation with cyclodextrins (CDs) was successfully applied [87]. Drug delivery of chrysin may also be enhanced with the use of carriers such as liposomes, micelles, and nanoparticles (nanostructured lipid carriers (NLCs), PLGA-PEG) [88]. In one study, nanostructured lipid carriers (NLCs) loaded with chrysin were used to inhibit the NRF2 pathway in MCF-7 cells [89]. It was shown that chrysin-loaded NLCs enhanced the percentage of apoptosis from 21.11 ± 5.72% to 27 ± 3.13% (*p* < 0.05). Furthermore, the population of cancer cells in the sub-G1 phase increased up to 12 ± 2.1% compared to untreated cells (*p* < 0.05). mRNA expression levels of NRF2, *NQO1*, HO1, and MRP1 exhibited a significant decrease compared to the control group (*p* < 0.05). NLCs, as a new generation of lipid-based drug delivery system, improved the efficacy of chrysin in sensitization of breast cancer MCF-7 cells to doxorubicin [89]. 2′,4′-dihydroxy-6′-methoxy-3′,5′-dimethylchalcone (DMC) was modified by conjugation with the amino acids L-alanine (compound **3a**) or L-valine (compound **3b**) to enhance anticancer activity and water solubility [90]. Compounds **3a** and **3b** had antiproliferative activity in human cervical cancer cell lines (C-33A, SiHa, and HeLa), with half-maximal inhibitory concentrations (IC_50_) of 7.56 ± 0.27 and 8.24 ± 0.14 µM, respectively, in SiHa cells. Moreover, compounds **3a** and **3b** inhibited SiHa cell migration in the wound healing assay. After treatment with compounds **3a** and **3b**, there was an increase in SiHa cells in the G1 phase, indicative of cell cycle arrest. Additionally, compound **3a** showed potential anticancer activity by upregulating TP53 and CDKN1A, which resulted in upregulation of BAX and downregulation of CDK2 and BCL2, leading to apoptosis and cell cycle arrest. The BAX/BCL2 expression ratio was increased after treatment with compound **3a** via the intrinsic apoptotic pathway. In silico molecular dynamics simulation and binding free energy calculation shed light on how these DMC derivatives interact with the HPV16 E6 protein, a viral oncoprotein associated with cervical cancer [90].

In recent years, another direction of research on overcoming cancer cell resistance has been developing, namely the use of plant-derived substances in photodynamic therapy (PDT) of MDR cells. Natural photosensitizers, such as curcumin or berberine, have properties similar to synthetic photosensitizers (PSs) [91]. It can be expected that, in the upcoming years, there will be more data on the effectiveness of natural PSs in anticancer therapy, including overcoming MDR.

## 6. Conclusions

Multidrug resistance of cancer cells is a common, unfavorable phenomenon occurring in patients undergoing chemotherapy. The NRF2 protein is one of the main factors responsible for the development of MDR. In many types of cancer, NRF2 is overexpressed, inhibiting the action of anticancer drugs. Therefore, NRF2 inhibitors may be new effective anticancer drugs, either as single compounds or in combination with already known drugs. So far, several substances of plant origin have been discovered that inhibit the activity of this protein. Some are being tested on in vivo models and undergoing clinical trials [92]. However, research on the possibility of overcoming MDR by inhibiting NRF2 with substances of plant origin is at an initial stage. These studies should be continued, especially since the plant kingdom is a huge source of biologically active compounds whose molecular mechanisms may meet the criteria of inhibiting NRF2 and preventing MDR. Natural compounds may become a starting point for the development of new anticancer drugs and strategies to overcome MDR in the future.
